# Transvaginal approach combined intracavitary and interstitial brachytherapy assisted by transrectal ultrasound: results from 30 patients with locally advanced cervical cancer

**DOI:** 10.1007/s11604-023-01481-4

**Published:** 2023-08-17

**Authors:** Takaaki Nakashima, Keiji Matsumoto, Tadamasa Yoshitake, Hiroaki Wakiyama, Osamu Hisano, Ryuji Uehara, Masanori Takaki, Takeshi Oshima, Hideaki Yahata, Kousei Ishigami

**Affiliations:** 1 Department of Clinical Radiology, Graduate School of Medical Sciences, Kyushu University, 3-1-1 Maidashi, Higashi-Ku, Fukuoka, 812-8582 Japan; 2 Department of Obstetrics and Gynecology, Graduate School of Medical Sciences, Kyushu University, Fukuoka, Japan

**Keywords:** Intracavitary and interstitial brachytherapy, Hybrid brachytherapy, Cervical cancer, Transvaginal approach, Transrectal ultrasound

## Abstract

**Purpose:**

This study evaluated the efficacy and safety of transvaginal approach combined intracavitary and interstitial brachytherapy (IC/IS BT) assisted by transrectal ultrasound (TRUS) for treatment of locally advanced cervical cancer (LACC).

**Materials and Methods:**

A total of 30 patients of LACC treated with external beam radiotherapy and IC/IS BT via transvaginal approach assisted by transrectal ultrasound were observed retrospectively. The 2-year local control (LC), progression-free survival (PFS), and overall survival (OS) were analyzed using the Kaplan–Meier method. Late adverse events were also evaluated to assess the safety of IC/IS BT.

**Results:**

The median follow-up period was 22 months. The 2-year LC, PFS, and OS were 90%, 61%, and 82%, respectively. We observed no critical complications related to the IC/IS BT technique. Late adverse events of grade 3 or more included one case of grade 4 colon perforation.

**Conclusion:**

Our patient series demonstrated that radiotherapy combined with transvaginal approach, TRUS-assisted IC/IS BT achieves favorable local control and safety for LACC.

## Introduction

Combined intracavitary and interstitial brachytherapy (IC/IS BT) enables tumor-specific dose escalation resulting in significantly higher local control of large tumors without adding treatment-related late morbidity in locally advanced cervical cancer (LACC) [1]. In Japan, the Japanese Society for Radiation Oncology (JASTRO) consensus guideline of IC/IS BT for gynecological cancers was published in 2021 and presented several needle-insertion techniques according to each insertion route and assisting modality [2]. At our department, IC/IS BT for LACC is usually performed by freehand needle insertion via transvaginal approach with transrectal ultrasound (TRUS) guidance. That is because deep sedation is not necessarily required in the transvaginal approach whereas it is with transperineal insertion. In addition, TRUS achieves clearer visibility and easier solo execution of all procedures of needle insertion compared with transabdominal ultrasound. Several clinical results have been reported showing the effectiveness of IC/IS BT in LACC [3, 4]. The international study on MRI-guided brachytherapy in cervical cancer (EMBRACE-I), which included patients treated with transvaginal approach IC/IS BT mainly based on MRI, presented a favorable efficacy, however, didn’t report needle insertion techniques in particular [3]. In addition, the study demonstrated that 14.6% of enrolled patients experienced Grade 3–5 late adverse events (genitourinary, gastrointestinal, vaginal, fistulas). In a prospective phase I/II clinical trial conducted in Japan, it was reported that IC/IS BT was performed using either a transvaginal or transperineal approach, and no difference in acute non-hematologic adverse events related to IC/IS BT was observed between the two approaches [4]. However, this study did not provide details on more specific needle-insertion techniques beyond the insertion route of the interstitial needle, nor did it examine potential differences in late adverse events and treatment effectiveness.

The purpose of this study was to evaluate the efficacy and safety of IC/IS BT in LACC patients using only the transvaginal needle insertion with TRUS guidance.

## Materials and Methods

This study was retrospectively performed at a single institution and was approved by our institutional ethical review board. A total of 30 patients with LACC who underwent primary radiotherapy with at least one session of IC/IS BT between April 2017 and December 2019 were included in this study. The indications for IC/IS BT at our institution are mainly for LACC tumors more than 4 cm in maximum diameter or having an asymmetrical shape, which cannot be delivered an adequate dose using conventional intracavitary brachytherapy (ICBT), given the dose constraints of surrounding organs at risk (OARs).

The standard radiotherapy regimen at our institution in LACC is shown in Table 1. External beam radiotherapy (EBRT) was delivered as three-dimensional conformal radiotherapy or intensity-modulated radiotherapy by a linear accelerator (TrueBeam or TrueBeam STx, Varian Medical System, Palo Alto, CA) with 10-MV photons. The clinical target volume (CTV) of EBRT included the gross tumor volume (GTV), whole uterus, parametria, upper part of the vagina, and regional lymph nodes (common iliac, external iliac, internal iliac, obturator, presacral nodes). In addition, paraaortic lymph node regions were covered in CTV in cases with metastases of common iliac and paraaortic lymph nodes. EBRT of 30.6 Gy in 17 fractions was delivered to the whole pelvis with a four-field box technique followed by 14.4 Gy in 8 fractions with the anterior–posterior/posterior–anterior parallel-opposed field technique, adding a central shield (CS) of 3–4 cm width to reduce the dose to the rectum and bladder. For patients with poor treatment response whose tumors had not shrunk enough to allow insertion of a tandem applicator at the end of 30.6 Gy EBRT, CS was added after whole-pelvis EBRT was delivered up to 39.6 Gy in 22 fractions. In cases of adenocarcinoma generally considered radioresistant, whole-pelvis EBRT was delivered up to 45 Gy in 25 fractions without CS. In patients with lymph node metastases, a median dose of 6 Gy in 3 fractions (range 6–16 Gy in 2–8 fractions) was additionally delivered. Chemotherapy was performed with 4–5 cycles of weekly cisplatin administration (40 mg/m^2^). Patients older than 75 years or with renal dysfunction or another disqualifying comorbidity were usually treated without chemotherapy.

**Table 1:** Radiotherapy regimen

EBRT		Brachytherapy	*n* = 30
WP	WP with CS		
30.6 Gy/17 fr	14.4 Gy/8 fr	24 Gy/4 fr	20
39.6 Gy/22 fr	5.4 Gy/3 fr	18 Gy/3 fr	4
39.6 Gy/22 fr	5.4 Gy/3 fr	24 Gy/4 fr	4
45.0 Gy/25 fr	–	24 Gy/4 fr	2

*EBR*T external beam radiotherapy, *WP* whole pelvis, *CS* central shield

Brachytherapy was performed with Fletcher-Williamson Asian Pacific metal tandem and ovoid applicators (Elekta AB, Stockholm, Sweden) by a ^192^Iridium remote afterloading system (RALS, MicroSelectron HDR™, Elekta AB, Stockholm, Sweden). In cases with extensive vaginal infiltration or a narrow vagina, the tandem cylinder applicator was indicated instead of tandem and ovoid applicators. Plastic or metallic interstitial needles were inserted via the transvaginal approach assisted by TRUS in all cases after tandem and ovoid applicators were inserted or before a tandem cylinder applicator was inserted. The interstitial needles were often inserted into the lateral side of tandem or ovoid applicators. In case it was necessary to insert the needles in both the ventral and dorsal sites of the tumors, it was often started from the ventral site. Plastic needles were often indicated because of the short offset; metallic needles were selected in cases of especially hard tumors. The intermittent intravenous administration of midazolam and analgesics (pentazocine or fentanyl) was taken under a continuous blood pressure monitor, pulse oximeter, and electrocardiogram during brachytherapy. All brachytherapy planning was based on CT images by Oncentra® (Elekta AB, Stockholm, Sweden) after fixation of applicators and interstitial needles at each session by reference to magnetic resonance images (MRI) obtained before radiotherapy began. Brachytherapy was performed once or twice a week after EBRT adding CS was initiated. Three or four brachytherapy sessions were conducted according to the total doses for OARs. High-risk CTV (HR-CTV) and OARs were contoured by reference to the guideline of the Japanese Radiation Oncology Study Group (JROSG) [5]. The relevant OARs include the rectum, sigmoid colon, small intestine, and bladder. The objective of the dosing regimen was to administer a dose of more than 6 Gy to the HR-CTV D90 (dose covering at least 90% of the HR-CTV) at each session, while simultaneously restricting the total dose delivered to the OAR D2 cm^3^ (dose at 2 cm^3^ of the OARs) to less than 70 Gy for the rectum, sigmoid colon, and small intestine and 80 Gy for the bladder. The total dose of EBRT and brachytherapy was calculated by a biologically equivalent dose in 2-Gy fractions (EQD2) according to the LQ model. The dose of EBRT adding CS was excluded. A value of *α*/*β* value was, respectively, assumed as 10 Gy for tumors and 3 Gy for OARs, in accordance with the Gynecological Groupe Européen de Curiethérapie-European Society for Radiotherapy and Oncology Study Group (GYN GEC-ESTRO) [6].

Local control (LC) of the primary lesion, progression-free survival (PFS), and overall survival (OS) were calculated from the initiation of radiotherapy using the Kaplan–Meier method with JMP® ver. 16 (SAS Institute, Cary, NC, USA). Patterns of recurrence were also assessed. In addition, late adverse events were evaluated using the National Cancer Institute Common Terminology Criteria for Adverse Events (CTCAE), version 4.0.

## Results

The characteristics of patients are presented in Table 2. Eighteen patients (60%) were International Federation of Gynecology and Obstetrics (FIGO) stage (2008) IIIB, and the median diameter of the tumors was 5.5 cm (range 3.3–8.7 cm). In 27 patients (90%), IC/IS BT was performed at all brachytherapy sessions. In others, two patients received IC/IS BT at 2 of 4 sessions due to tumor shrinkage, and one received only 1 of 4 planned sessions. The median number of interstitial needles used for IC/IS BT was 1 (range 1–9).

**Table 2:** Patient characteristics

	*n* = 30
Age (median, range) (years)	61.5 (range 31–82)
Histology	
Squamous cell carcinoma	28
Adenocarcinoma	2
FIGO stage (2008)	
IIB	10
IIIB	18
IVA	2
Pelvic lymph node metastasis	
+	12
−	18
Paraaortic lymph node metastasis	
+	1
−	29
Tumor diameter (median, range) (cm)	5.5 (range 3.3–8.7)
Concurrent chemotherapy	
+	24
−	6

*FIGO* International Federation of Gynecology and Obstetrics

The median volume of HR-CTV at the first session of IC/IS BT was 41.9 cm^3^ (range 11–140 cm^3^). The median total dose of HR-CTV D90 in EQD2 was 76.9 Gy (range 68–101 Gy). Median total doses of OARs D2 cm^3^ in EQD2 were 63.8 Gy (range 53–80 Gy) to the rectum, 55.2 Gy (range 33–71 Gy) to the sigmoid colon, 54.3 Gy (range 35–77 Gy) to the small intestine, and 72.1 Gy (range 53–93 Gy) to the bladder (Table 3).

**Table 3:** Summary of parameters in dose–volume histogram

	Median (range)	Planning objective
CTV HR volume	41.9 cm^3^ (11–140)	
Total dose of CTV HR (D90)	76.9 Gy (68–101)	≥ 60 Gy
Total dose of rectum (D2cm^3^)	63.8 Gy (53–80)	< 70 Gy
Total dose of sigmoid colon (D2cm^3^)	55.2 Gy (33–71)	< 70 Gy
Total dose of small intestine (D2cm^3^)	54.3 Gy (35–77)	< 70 Gy
Total dose of bladder (D2cm^3^)	72.1 Gy (53–93)	< 80 Gy

*CTV HR* high-risk clinical target volume

**Fig. 1:**
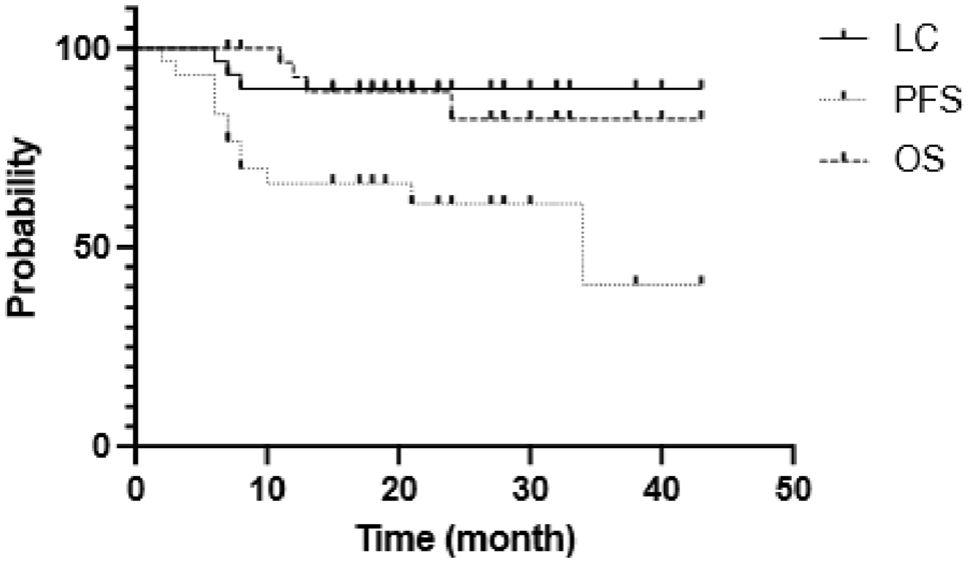
Kaplan–Meier survival curve for local control (LC), progression-free survival (PFS), and overall survival (OS) of the primary lesion in locally advanced cervical cancer (LACC) treated with radiotherapy including combined intracavitary and interstitial brachytherapy (IC/IS BT)

**Fig. 2:**
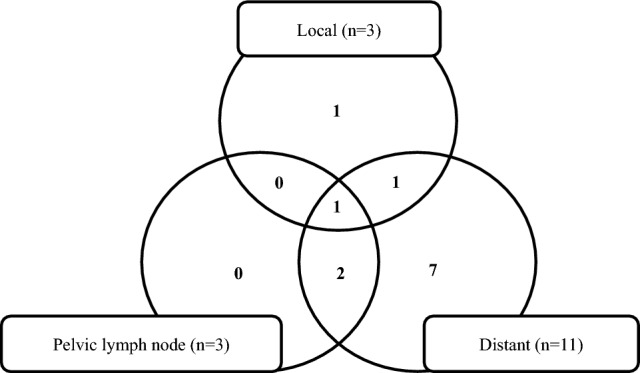
Pattern of recurrence. The Venn diagram shows the number of each recurrence pattern. Distant metastases were most frequent, with only one case of local recurrence

Median follow-up was 22 months (range 7–43 months). The 2-year LC, PFS, and OS were 90%, 61%, and 82%, respectively (Fig. 1). Three patients developed local recurrence of the primary lesion, three patients developed lymph node recurrence, and 11 patients developed distant metastasis (Fig. 2). Sites of distant metastases were as follows: lung (*n* = 7), paraaortic lymph node (*n* = 6), mediastinal lymph node (*n* = 1), peritoneal dissemination (*n* = 2), bone (*n* = 1), and spleen (*n* = 1); some cases had more than one site of metastasis.

No patients suffered from complications related to the IC/IS BT technique, such as injury of adjacent organs, severe infection, or bleeding requiring transcatheter arterial embolization. Late adverse events included grade 1 rectal bleeding in 4 patients, grade 4 perforation of the sigmoid colon in 1 patient, and grade 1 hematuria in 2 patients (Table 4). Only one case suffered from perforation of the sigmoid colon about 11 months after the completion of radiotherapy and eventually required urgent operation. In this case, the total dose to the sigmoid colon D2 cm^3^ in EQD2 was 53.9 Gy. This case suffered from a recurrence of distant metastases without local recurrence of the primary lesion about seven months after the completion of radiotherapy and received chemotherapy, including two cycles of bevacizumab.

**Table 4:** Summary of late adverse events

	*n* (%)	Late adverse events
Gastrointestinal		
Grade 1	4 (13%)	Rectal bleeding
Grade 2	0 (0%)	
Grade 3	0 (0%)	
Grade 4	1 (3%)	Perforation of colon
Urinary		
Grade 1	2 (6%)	Hematuria
Grade 2	0 (0%)	
Grade 3	0 (0%)	
Grade 4	0 (0%)	

## Discussion

In the present study, the 2-year LC rate was 90% despite the tumor diameter and volume of HR-CTV being relatively large, and most patients being relatively advanced, at FIGO stage IIIB or more. The clinical outcomes were comparable with previous investigations in which IC/IS BT was performed in all cases of LACC (Table 5) [7]. In addition, although many patients in the present study had large tumor volumes and advanced-FIGO-stage LACC, the results were comparable to those of other studies in which intracavitary brachytherapy without insertion of interstitial needles was applied to all patients [8, 9]. The retroEMBRACE study reported that local recurrences of primary lesions developed frequently within two years of follow-up [10], so it was important to consider the 2-year LC of primary lesions in our series.

**Table 5:** Present study and previous studies on radiotherapy including intracavitary brachytherapy for cervical cancer

	FIGO stage (%)	Tumor diameter (cm) Median (range)	CTV HR volume (cm^3^) Median (range)	CTV HR D90 (Gy) Median (range)	2-year LC (%)	Late adverse events (Grade ≥ 3) *n* (%)
Murakami et al. (*n* = 42) [7]	≤ IIIA: 26 ≥ IIIB: 74	6.0 (3.9–10.1)	37 (12–89)	70 (56–97)	80	3 (7%) G3 rectal bleeding: 2 G3 rectovaginal fistula:1
Kusada et al. (*n* = 68) [8]	≤ IIIA: 70 ≥ IIIB: 30	4.6 (2.4–9.3)	28 (10–128)	72 (55–95)	83	6 (8.8%) G3 colitis: 4 G4 colitis: 2
Present study (*n* = 30)	≤ IIIA: 33 ≥ IIIB: 67	5.5 (3.3–8.7)	42 (11–140)	76 (68–101)	90	1 (3.3%) G4 perforation of colon

*FIGO* International Federation of Gynecology and Obstetrics, *CTV HR* high-risk clinical target volume, *LC* local control

With respect to the total dose of HR-CTV D90 in EQD2, our department sets a goal of 60 Gy or more. The median dose in the present study was 76.9 Gy, and the goal was achieved in all cases. Murakami et al. reported that LC was favorable in cases that achieved 60 Gy in EQD2 or more of HR-CTV D90 [11]. On the other hand, the EMBRACE-II study adopted a planning aim for an HR-CTV D90 of 90 Gy in EQD2, and the limit for the prescribed dose in EQD2 was 85 Gy [12]. The EMBRACE-I, which enrolled patients treated with IC/IS BT, presented a favorable 5-year LC of 92% in the subgroup of FIGO stage IIIB LACC patients whose tumors had a median HR-CTV volume of 40 cm^3^ and were delivered a median dose of 88 Gy in EQD2 for HR-CTV D90 [3]. Because the EMBRACE study did not use a central shield (CS) in whole-pelvis EBRT and generally indicated image-guided brachytherapy based on MRI, it was impossible to strictly compare clinical outcomes with those of the present study. Considering the dose contribution in the field blocked by CS of whole-pelvis EBRT reported by Tamaki et al. [13], however, the total dose of HR-CTV D90 in the present study was lower than that of the EMBRACE study. In the present study, all three patients suffering from a local recurrence of primary lesions were characterized by squamous cell carcinoma and FIGO stage IIIB, and their tumor diameter at the initiation of radiotherapy was 6 cm or more (Table 6). In a recent multicenter prospective clinical study of CT-based intracavitary brachytherapy in Japan employing dose calculations excluding the dose blocked by CS, the median total dose for HR-CTV D90 was 70 Gy in EQD2 [14]. Given the results of the present study, however, it is possible that it is necessary to escalate the total dose of HR-CTV D90 in larger tumors. There are only limited data demonstrating useful indicators except for HR-CTV D90. The previous retrospective studies reported that other indicators, such as HR-CTV D98 and V200, were associated with improved local control [7, 15]. To achieve the dose escalation and more favorable dose coverage of the tumor, it might be also useful to increase the number of needles inserted and introduce IC/IS BT of the transvaginal approach combined with the transperineal approach or pure interstitial brachytherapy. In addition, brachytherapy planning based on real-time MRI imaging can obtain more definite contouring of each organ and sophisticate the dose optimization than those based on CT imaging.

**Table 6:** Details of cases with local recurrence

Case	Histology	FIGO stage	Tumor diameter (cm)	HR-CTV volume (cm^3^)	HR-CTV D90 (Gy)	Site of recurrence	Late adverse events (Grade ≥ 3)
1	SCC	IIIB	6.3	43.8	76.3	Uterus, parametria, pelvic and paraaortic lymph nodes	–
2	SCC	IIIB	6.5	28.2	78.7	Uterus, parametria	–
3	SCC	IIIB	7.2	109.6	68.0	Uterus, paraaortic lymph nodes, lung	–

*SCC* squamous cell carcinoma, *FIGO* International Federation of Gynecology and Obstetrics, *HR-CTV* high-risk clinical target volume

With respect to the technique in IC/IS BT, the transvaginal approach applied in all cases of the present study seems to be easier and safer than the transperineal one mainly because the route to the tumor is shorter, and the procedure does not require as high a degree of sedation as saddle block [2]. In addition, the insertion of interstitial needles was performed by TRUS in all cases of the present study mainly because TRUS achieves clearer visibility and easier solo execution of all procedures of needle insertion under real-time guidance with lesser X-ray exposure compared with transabdominal ultrasound and CT. No patients suffered from injury of adjacent organs, severe infection, or bleeding requiring transcatheter arterial embolization. Late adverse events of grade 3 or more were no more frequent in our study than in previous studies (Table 5). Therefore, IC/IS BT performed with the transvaginal approach assisted by TRUS could be achieved safely. As the limitations of TRUS, it might be necessary to keep in mind that there are some patients in which TRUS could not always achieve an apparent view near the site of needles inserted partly because of individual anatomy and intestinal gas. In addition, insertion of the probe sometimes causes pain and discomfort in TRUS compared with transabdominal ultrasound.

We experienced one case of sigmoid colon perforation as a serious adverse event. Because the total dose to the sigmoid colon of this case was 53.9 Gy (D2 cm^3^ in EQD2), which was within the planning objective, the administration of bevacizumab after completion of radiotherapy was thought to be one of the possible causes. A previous investigation reported that the administration of bevacizumab might increase the risk of severe adverse events in LACC cases treated with radiotherapy [16]. Therefore, radio-oncologists need to remember the possibility of bevacizumab being administered in cases suffering from recurrence and continue to reduce doses to OARs as low as possible in radiotherapy treatment planning.

We acknowledge that this study has several limitations, including its retrospective nature, single-institutional design, and small sample size. Therefore, it is important to accumulate a larger number of cases in future studies to further validate our findings.

In conclusion, the present study presented that radiotherapy with IC/IS BT using the transvaginal approach assisted by TRUS for LACC could achieve favorable local control and safety. Transvaginal approach does not necessarily require deep analgesia and appears to be less invasive for patients.
